# Fetal Cytokine Balance, Erythropoietin and Thalassemia but Not Placental Malaria Contribute to Fetal Anemia Risk in Tanzania

**DOI:** 10.3389/fimmu.2021.624136

**Published:** 2021-04-30

**Authors:** Edward R. Kabyemela, Michal Fried, Jonathan D. Kurtis, Gwamaka Moses, J. Patrick Gorres, Atis Muehlenbachs, Patrick E. Duffy

**Affiliations:** ^1^ Mother Offspring Malaria Studies (MOMS) Project, Seattle Biomedical Research Institute, Seattle, WA, United States; ^2^ School of Medicine, Muhimbili University of Health and Allied Sciences, Dar es Salaam, Tanzania; ^3^ Laboratory of Malaria Immunology and Vaccinology, National Institutes of Allergy and Infectious Diseases, National Institutes of Health (NIH), Bethesda, MD, United States; ^4^ Department of Pathology and Laboratory Medicine, Center for International Health Research, Rhode Island Hospital, Brown University, Providence, RI, United States; ^5^ Mbeya College of Health and Allied Sciences, University of Dar es Salaam, Mbeya, Tanzania

**Keywords:** placental malaria, fetal anemia, thalassemia, cytokines, erythropoietin

## Abstract

Fetal anemia is common in malaria-endemic areas and a risk factor for anemia as well as mortality during infancy. Placental malaria (PM) and red cell abnormalities have been proposed as possible etiologies, but the relationship between PM and fetal anemia has varied in earlier studies, and the role of red cell abnormalities has not been studied in malaria-endemic areas. In a Tanzanian birth cohort study designed to elucidate the pathogenesis of severe malaria in young infants, we performed a cross-sectional analysis of risk factors for fetal anemia. We determined PM status, newborn red cell abnormalities, and maternal and cord blood levels of iron regulatory proteins, erythropoietin (EPO), cytokines and cytokine receptors. We examined the relationship between these factors and fetal anemia. Fetal anemia was present in 46.2% of the neonates but was not related to PM. Maternal iron deficiency was common (81.6%), most frequent in multigravidae, and interacted with parity to modify risk of fetal anemia, but it was not directly related to risk. Among offspring of iron-deficient women, the odds of fetal anemia increased with fetal α^+^-thalassemia, as well as these patterns of cord blood cytokines: increased cord IL-6, decreased TNF-RI, and decreased sTfR. The EPO response to fetal anemia was low or absent and EPO levels were significantly decreased in newborns with the most severe anemia. This study from an area of high malaria transmission provides evidence that 1) fetal α^+^-thalassemia and cytokine balance, but not PM at delivery, are related to fetal anemia; 2) maternal iron deficiency increases the risk that other factors may cause fetal anemia; and 3) fetal anemia has a multifactorial etiology that may require a variety of interventions, although measures that reduce maternal iron deficiency may be generally beneficial.

## Introduction

Fetal anemia can be the result of immune or non-immune insults. The most common immune etiology is maternal Rhesus (Rh) disease. If untreated, fetal anemia may lead to hydrops, multi-organ failure and fetal death (1, 2). In Malawi, fetal anemia at birth increased anemia risk during the first half of infancy, as well as shorter time to first illness, higher cumulative morbidity, and infant mortality (3, 4). In Kenya, anemia during infancy is a risk factor for infant mortality (5).

Fetal anemia, defined as cord blood hemoglobin less than 12.5g/dl, is common in areas where malaria is transmitted (6). Placental malaria (PM) and thalassemia are also common in areas of stable transmission, and have been proposed but not proven to explain the high incidence of fetal anemia in such areas (6). The association of fetal anemia and PM has varied in earlier studies with some showing association (7–9) but others reporting no association (10–12). In an area of high parasite resistance to sulfadoxine-pyrimethamine (SP) in Tanzania, the use of SP for intermittent preventive therapy (IPTp) to prevent PM and the parasite *dhps* c581 SP resistance allele are independently related to decreased levels of cord blood hemoglobin in the newborn (13).

α^+^-thalassemia has not been examined as a cause of fetal anemia in areas of malaria transmission (6). Because malaria is rare in African newborns (14), hemolysis of infected red cells cannot explain fetal anemia, but other mechanisms related to malarial anemia could be involved. In older children and adults, malarial anemia has been associated with inflammatory cytokines like tumor necrosis factor-α (TNF-α) and interferon-γ (IFN-γ) that can inhibit erythropoiesis (15), cause dyserythropoiesis and promote erythrophagocytosis (16). Levels of the anti-inflammatory cytokine interleukin (IL)-10 (17) and the ratio of IL-10 to TNF-α (18) are significantly lower in African children during severe malarial anemia, and placental levels of TNF-α are significantly higher in African mothers with severe malarial anemia (19).

Iron deficiency is another major cause of anemia, is common among pregnant women living in resource-poor settings of both malarious and non-malarious countries, and is more common in multigravid than primigravid women (20, 21). Neonatal iron stores are influenced by maternal iron storage levels (22) and maternal iron deficiency anemia was found to be a risk factor for fetal anemia in Malawi (7). Transplacental transfer of iron from the mother is the only source of iron to the developing fetus. This is achieved through the formation of an iron-transferrin complex (Fe-Tf), with iron from maternal circulation binding to transferrin receptor 1 (TFR1) on the apical surface of the syncytiotrophoblast (23). This complex is endocytosed, then iron is released into the endosome through ferroportin on the basal side of the syncytiotrophoblast, and finally into fetal circulation (24).

Erythropoietin (EPO) is a major regulator of RBC production in the bone marrow. EPO binds to its receptor on the red blood cell (RBC) progenitor surface, inducing proliferation and terminal differentiation of erythroid precursor cells while also protecting against RBC precursor apoptosis (25). Several studies have reported increases in cord blood EPO concentration in response to fetal hypoxia caused by a variety of conditions including intrauterine growth restriction (26) and maternal smoking (27). Increased levels of EPO in blood or amniotic fluid have also been reported in neonates with severe anemia caused by hemolytic anemia (28) and Rh immunization (29).

In the present study, we searched for potential causes and mediators of fetal anemia in an area of Tanzania with intense malaria transmission. We determined PM status, iron status in mothers and newborns, and inherited red cell abnormalities in newborns. We measured plasma levels of inflammatory and anti-inflammatory cytokines that have been previously implicated in the pathogenesis of anemia (TNF-α and its receptors (soluble TNF-RI and TNF-RII), IFN-γ, IL-6, and IL-10) in cord blood. Levels of sTfR and erythropoietin (EPO) were also measured in these samples. We examined the relationship of all these factors to the risk of fetal anemia.

## Materials and Methods

### Study Cohort and Laboratory Procedures

Mothers and newborns included in this analysis were participating in a birth cohort study known locally as the Mother-Offspring Malaria Studies (MOMS) Project at Muheza Designated District Hospital, Muheza, Tanga-Tanzania. The study protocols were approved by the Division of Microbiology and Infectious Diseases at the U.S. National Institutes of Health and by the institutional review boards of Seattle Biomedical Research Institute (contracted to the Western Institutional Review Board, Puyallup, WA, USA, now WCGH IRB) and the Medical Research Coordinating Committee in Tanzania. Written informed consent was obtained from each child’s mother before participation for herself and her newborn. Clinical procedures for the MOMS Project have been previously described (30–38). Hemoglobin was measured using a Cell Dyne 1200 hematology analyzer (Abbot Diagnostics Division, Abbot Park, IL-60064, USA).

Maternal peripheral blood was obtained by venipuncture from women immediately after delivery and anticoagulated with citrate phosphate dextrose. Plasma was obtained by centrifugation at 3,000 *g* for 3 min and stored at -70°C until thawed on the day that assays were performed. Thick and thin smears were prepared; thin smears were fixed with methanol. Blood slides were stained for 10 minutes in 10% Giemsa, washed in tap water, air-dried, then examined using light microscopy at 100 × magnification. Ten thousand red cells were examined in the thin smear before concluding that a placental blood slide was negative.

Levels of ferritin, sTfR, EPO, cytokines and cytokine receptors were analyzed using a multiplexed, bead-based platform (BioPlex^®^, BioRad, Irvine, CA) and custom-made assay kits as previously described (39, 40). Detection limits for these assays were as follows: ferritin - 0.07 ng/ml, soluble transferrin receptor (sTfR) - 0.03 ng/ml, EPO - 0.1 mIU/ml, TNF-α - 0.1 pg/ml, TNF receptor (R) I - 1.58 pg/ml, TNF-RII - 0.21 pg/ml, IFN-γ - 0.04 pg/ml, IL-6 - 1.45 pg/ml, IL-10 - 0.02 pg/ml. Levels of soluble factors were adjusted to account for dilution in anticoagulant at the time of sample collection. For each plasma sample, all analytes were assayed in a single day, thus eliminating freeze/thaw cycles.

Sickle cell variants (HbAA, HbAS and HbSS) were determined by cellulose acetate paper electrophoresis according to the manufacturer’s instructions (Helena Laboratories, Beaumont, Texas, USA). Genotyping for α^+^-thalassemia and glucose-6-phosphate dehydrogenase (G6PD) variants was performed by polymerase chain reaction (PCR) techniques. DNA was isolated from blood spots on filter paper according to the manufacturer’s instructions (Generation^®^ Capture Card Kit, Gentra Inc.). α^+^-thalassemia typing was done according to the protocol described by Chong et al. (41).

To type G6PD variants (G6PD B, G6PD A, and G6PD A-), a region spanning the third to fifth exon was amplified by an outer PCR (Forward 5’- GGT GGA TGA TGT ATG TAG-3’ and Reverse 5’- GCA ACG CTG CCA CCT TGT G-3’), followed by nested multiplex PCR. Nested primers to detect the residue 202 polymorphism were G6PD-202 F (5’-CCT TCT GCC CGA AAA CAC CTT CACC-3’) and G6PD-202 R (5’- GTC CCC GAA GCT GGC CAT GCT GG -3’); and primers to detect the residue 376 polymorphism were G6PD-376 F (5’- TAC CAG CGC CTC AAC AGC CCC ATG –3’) and G6PD-376 R (5’- GGA CTC GTG AAT GTT CTT GGT GAC G-3’). The PCR conditions for the first reaction were as follows: samples were subjected to initial denaturation for 60 seconds at 94°C followed by 30 cycles of 94°C for 60 seconds, 58°C for 45 seconds and 72°C for 60 seconds, followed by 7 minutes at 72°C. The nested multiplex PCR conditions were similar to the above except the annealing temperature was set at 56°C. Mismatches in the primers (at the underlined residues) introduced NcoI digestion sites into the amplified products of the variant alleles, leading to digestion of the G6PD-202 product in G6PD A individuals and digestion of the G6PD-376 product in G6PD A- individuals.

### Clinical and Parasitological Definitions

Maternal anemia was defined as maternal venous blood hemoglobin concentration less than 11.0 g/dl. Fetal anemia was defined as cord blood hemoglobin concentration less than 12.5 g/dl (6). Placental malaria status was determined from thick and thin smear of mechanically extracted placental blood, and the presence of placental parasites, inflammation and pigment in PM+ women was characterized by histology as previously described (42). Iron deficiency was defined as ferritin concentration <30 ng/ml when C-reactive protein (CRP) was ≤8.2 µg/ml (iron deficiency in the absence of inflammation), or ferritin concentration <70 ng/ml when CRP was >8.2 µg/ml (iron deficiency in the presence of inflammation) (42).

### Statistical Analysis

Analyses were performed using Statview 5.0.1 (SAS Institute, Cary, NC, USA). Differences between proportions were compared by χ^2^ test. Normally distributed continuous data were compared by the Student’s *t* test and analysis of variance. Data that did not conform to a normal distribution were compared by Mann-Whitney or Kruskal-Wallis tests. Simple and multiple logistic regression models were used to test for associations of maternal and fetal factors to fetal anemia. Two-sided p ≤0.05 was considered to be statistically significant.

In a stepwise approach (see **Supplementary Figure S1**), we searched for maternal and fetal factors related to fetal anemia. We first used a Chi-square test for categorical variables and a Student’s t-test for continuous variables to examine their direct relationship to fetal anemia. Because parity was strongly related to fetal anemia risk in that analysis, and is known to impact numerous pregnancy outcomes in malarious areas, we used ANOVA to examine the interactions of parity with other baseline maternal and fetal factors for their relationships to cord hemoglobin level. Secondly, we employed a simple logistic regression analysis to identify fetal soluble factors (iron regulatory proteins, erythropoietin, cytokines and cytokine receptors) related to fetal anemia. We then examined maternal and fetal factors that were seen to be significant in univariate analysis, for their independent association with fetal anemia in multiple logistic regression analysis. Finally, because the interaction term parity*maternal iron deficiency was related to cord hemoglobin level, we stratified the multivariate logistic regression analysis by maternal iron deficiency, and then by parity groups among mothers with iron deficiency.

## Results

### Fetal Anemia Is Related to Parity but Not to Maternal Anemia

We determined the hemoglobin level in 610 cord blood and 658 maternal blood samples (90 with PM) obtained at delivery. The overall prevalence of fetal anemia defined as cord hemoglobin below 12.5g/dL (6) was 46.2% and increased with successive pregnancies. 95.7% of deliveries were spontaneous vaginal deliveries without any recorded complications of labor. 26/610 (4.3%) were delivered *via* C-section, and 8/26 (30.8%) C-sections had fetal anemia. Mean (SD) cord hemoglobin (g/dL) was 12.5 (2.7), 12.6 (2.3) and 12.1 (3.0) in offspring of primigravidae, secundigravidae and multigravidae, respectively. Fetal anemia was present in 38.9%, 41.3% and 63.3% of first, second and later offspring, respectively (χ^2^=9.4, p = 0.008). Cord hemoglobin tended to be inversely correlated with increasing parity although this relationship was not statistically significant (r = - 0.071; p = 0.07).

Most women (356/658 (57.1%)) were anemic at delivery (hemoglobin < 11 g/dL), and this did not differ by parity (54.6%, 56.5% and 58.9% (χ^2^ = 0.93, p = 0.62) in primigravidae, secundigravidae and multigravidae respectively). The proportion of infants with fetal anemia did not differ significantly among offspring of mothers with anemia versus offspring of mothers without anemia (50.6% versus 43.6%; χ^2^ = 2.7, p = 0.1). This pattern was similar in the different parity groups (primigravid, 46.2% versus 36.6%, χ^2^ = 1.4, p = 0.2; secundigravid, 44.7% versus 41.3%, χ^2^ = 0.14, p = 0.7; multigravid, 55.6% versus 49.1, χ^2^ = 1.1, p = 0.3). When treated as a continuous variable, maternal and fetal hemoglobin were related, but the correlation was weak (Linear correlation coefficient = 0.15; p = 0.04)

### Fetal Anemia Is Not Related to Placental Malaria (PM) at Delivery

Fetal anemia was present in 240/520 (46.1%) offspring of PM- versus 49/90 (46.4%) offspring of PM+ mothers (p = 0.9). Although fetal anemia was more frequent in the PM+ than the PM- group in each parity category, none of these differences were statistically significant, using cord hemoglobin of either 12.5 g/dL (**Supplementary Table S1**) or of 10.0 g/dL (**Supplementary Table S2**) to stratify the population. Cord hemoglobin levels did not differ significantly between offspring of PM+ (mean (SD) 12.0 (2.9) g/dL) versus PM- women (mean (SD) 12.3 (2.7) g/dL). Mean (SD) cord hemoglobin levels were 12.3 (2.8) g/dL and 12.2 (2.8) g/dL in offspring of PM+ mothers with inflammation and PM+ mothers without inflammation respectively. The proportions of newborns with fetal anemia did not differ significantly between PM- (46.1%), PM+ without placental inflammation (46.4%), and PM+ with placental inflammation (57.6%), defined histologically as presence of intervillous inflammatory cells (χ^2^ = 1.3, p = 0.51). The proportion with fetal anemia did not differ between offspring of mothers with active, past or no placental malaria by histology (p = 0.8).

### Maternal Iron Status and Parity Interact to Modify Risk of Fetal Anemia

Parity was significantly related to fetal anemia (**Table 1**) and is well-known to impact numerous maternal and fetal outcomes in malarious areas, including its strong effect on placental malaria risk as women acquire protective immunity over successive pregnancies (43). We therefore examined whether the interaction of parity with other maternal and fetal baseline factors was related to cord hemoglobin levels, as an indication whether these interactions might be confounding analyses of fetal anemia risk. Among all interactions examined (**Supplementary Table S3**), only the interaction of parity with maternal iron status was significantly related to cord blood hemoglobin levels (factorial ANOVA, p = 0.03), suggesting that fetal anemia risk factors should be examined after stratification by maternal iron status and parity.

**Table 1:** Maternal and newborn characteristics in relation to fetal anemia*.

Characteristic	Fetal Anemia	No Fetal Anemia	P value
**MATERNAL**			
Age (Years), Mean (SD)	26.46 (6.3)	25.25 (6.0)	**0.02**
Iron deficiency (n = 461)	211 (45.8%)	250 (54.2%)	0.8
Anemia (n = 314)	159 (50.6%)	155 (49.4%)	0.1
**Parity**			
Primigravidae (n = 167)	65 (38.9%)	102 (61.1%)	**0.009**
Secundigravidae (n = 146)	61 (41.8%)	85 (58.2%)	
Multigravidae (n = 297)	156 (52.5%)	141 (47.5%)	
**PM (Histology) (n=610)**			
No infection (n = 498)	227 (45.6%)	271 (54.4%)	0.8
Active infection (n = 78)	38 (48.7%)	40 (51.3%)	
Past infection (n = 34)	17 (50.0%)	17 (50.0%)	
**PM (Microscopy)**			
PM+ (n = 90)	42/90 (46.7%)	48/90 (53.3%)	0.9
PM– (n = 520)	240/520 (46.2%)	280/520 (53.8%)	
**NEWBORN**			
Birth Weight (g), Mean (SD)	3162 (0.41)	3203 (0.43)	0.3
Female/Male (n)	157/171	142/139	0.6
Iron deficiency (n =73)	33 (45.2%)	40 (54.8%)	1.0
**Sickle hemoglobin**			
AA (n = 488)	232 (84.0%)	256 (80%)	0.4
AS (n = 103)	42 (15.3%)	61 (19.1%)	
SS (n = 5)	2 (0.7%)	3 (0.9%)	
**G6PD status**			
A (n = 72)	35 (12.65%)	37 (11.7%)	0.2
A- (n = 72)	27 (9.7%)	45 (14.2%)	
A Heterozygous (n =52)	24 (8.6%)	15 (4.7%)	
A- Heterozygous (n = 39)	22 (8.0%)	30 (9.4%)	
B (n = 361)	170 (61.1%)	191 (60.0%)	
**Thalassemia**			
αα/ αα (n = 247)	104 (44.9%)	143 (54.2%)	**0.0005**
−α/ αα (n = 199)	92 (39.6%)	107 (40.5%)	
−α/ −α (n = 50)	36 (15.5%)	14 (5.3%)	

All variables were analyzed by Chi-square t-test except for maternal age and birth weight (unpaired t-test). Bold values represent significant differences.

Among mothers with available measurements, 81.6% (618/757) were iron-deficient, and iron deficiency was less common in primigravid (76.6%) than in secundigravid (85.7%) or multigravid women (82.7%; χ^2^ = 5.9, p = 0.05). By comparison, only 12.2% (93/726) of newborns had iron deficiency and this did not differ significantly by parity (12.2% in first, 10.2% in second, and 14.3% in third or later offspring; χ^2^ = 1.6, P = 0.4). The frequency of maternal or fetal iron deficiency did not differ between newborns with or without fetal anemia, whether defined as hemoglobin below 12.5 g/dL (**Table 1**) or below 10 g/dL (**Supplementary Tables S4** and **S5**).

**Figure 1:**
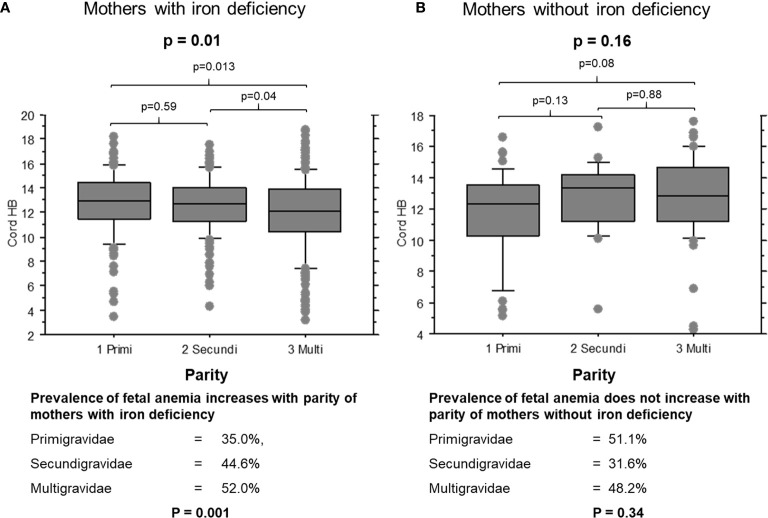
Cord blood hemoglobin levels in offspring of mothers with and without iron deficiency, stratified by parity. **(A)** Cord samples from mothers with iron deficiency: 1, primigravidae (n = 53); 2, secundigravidae (n = 72); 3, multigravidae (n = 132). **(B)** Cord samples from mothers with normal iron status: 1, primigravidae (n = 29); 2, secundigravidae (n = 14); 3, multigravidae (n = 31). The box plots indicate the median (horizontal line) and interquartile range (box), and the whiskers represent 10^th^/90^th^ percentiles. The differences between groups were analyzed by the Kruskal-Wallis test. Associated P values are shown.

When mothers were iron-deficient, cord hemoglobin levels decreased (**Figure 1A**, p = 0.01) and the prevalence of fetal anemia increased over successive pregnancies (35.0%, 44.6% and 52.0% of first, second and later offspring, respectively; χ^2^ = 8.9, p = 0.001). The opposite pattern was observed when mothers had adequate iron stores: cord hemoglobin levels increased (**Figure 1B**, p = 0.15) and the prevalence of fetal anemia decreased with increasing parity, although this was not statistically significant (51.1%, 31.6% and 42.8% of first, second and later offspring, respectively; χ^2^ = 2.1, p = 0.34).

### Thalassemia but No Other Red Cell Disorder Is Associated With Fetal Anemia

Samples from 496 newborns were genotyped for α^+^-thalassemia, 596 for G6PD deficiency and 596 for sickle cell hemoglobin phenotype. The frequency of neonatal genotypes/phenotypes did not vary by PM or by parity (**Supplementary Table S6**).

α^+^-thalassemia but not G6PD deficiency nor sickle hemoglobin increased fetal anemia risk. Overall, thalassemia homozygous (−α/ −α) offspring had the lowest hemoglobin levels (mean (SD) g/dL of 12.5 (2.9), 12.4 (2.7) and 10.9 (2.1) in normal, heterozygous or homozygous offspring; Kruskal Wallis test, p < 0.0001) and the highest frequency of fetal anemia (**Table 1**) compared to heterozygous (−α/ αα) or normal newborns.

Thalassemia significantly decreased fetal hemoglobin levels in offspring of multigravidae but not in offspring of primigravidae or secundigravidae. Mean (SD) cord hemoglobin (g/dL) was 12.5 (3.0), 12.4 (2.8) or 10.4 (1.8) in normal, heterozygous or homozygous offspring, respectively, of multigravidae (Kruskal-Wallis test, p = 0.0002), versus 12.4 (2.7), 12.4 (3.0) or 12.4 (2.7) in those of primigravidae (Kruskal-Wallis test, p = 0.86) and 12.7 (2.7), 12.8 (2.0) or 11.4 (2.0) in those of secundigravidae (Kruskal-Wallis test, p = 0.06). Thalassemia increased the frequency of fetal anemia in offspring of multigravidae (χ^2^ = 5.64, p = 0.02) but not primigravidae (χ^2^ = 0.001, p = 0.9) or secundigravidae (χ^2^ = 0.56, p = 0.45).

### Cytokine Balance, sTfR and Fetal Thalassemia Are Associated With Fetal Anemia Risk in Univariate and Multivariate Analyses

We performed a simple logistic regression analysis of cord soluble factors and risk of fetal anemia. Higher levels of TNFRII and IL-6 increased the risk of fetal anemia, whereas higher levels of TNF-RI and sTfR decreased the risk (**Table 2**). In an un-stratified multiple logistic regression analysis of these significant factors, −a/−a thalassemia and higher levels of IL-6 and TNF-RII independently increased, whereas higher levels of TNF-RI and sTfR decreased, the risk of fetal anemia (**Table 3**). Since maternal iron deficiency interacted with parity to modify the levels of cord blood hemoglobin, we stratified the multiple regression analysis by maternal iron status (**Table 4**). All relationships between newborn risk factors and fetal anemia occurred in the maternal iron deficiency group and were absent in the group with normal maternal iron status.

**Table 2:** Simple logistic regression analysis of soluble fetal factors in relation to fetal anemia.

FETAL FACTOR	OR	95% CI	P value
TNF-α (n = 576)	0.80	(0.49 - 1.21)	0.3
TNF-RI (n = 583)	0.46	(0.23 - 0.90)	**0.02**
TNF-RII (n = 583)	2.19	(1.24 - 3.90)	**0.007**
IFN-γ (n = 121)	1.71	(0.82 - 3.56)	0.2
IL-10 (n = 523)	1.08	(0.63 - 1.85)	0.8
IL-6 (n = 476)	1.62	(1.21 - 2.17)	**0.001**
sTfR (n = 583)	0.30	(0.14 - 0.62)	**0.001**
EPO (n = 402)	1.40	(0.78 - 2.52)	0.3

Bold values represent significant differences.

**Table 3:** Multiple logistic regression analysis of factors in relation to fetal anemia risk (n = 358).

FACTOR	aOR*	95% CI	P value
Secundigravidae	0.41	0.12-1.47	0.2
Multigravidae	0.27	0.02-2.72	0.3
Maternal iron deficiency	0.29	0.07-1.26	0.1
Maternal iron deficiency * Parity	1.63	0.89-3.08	0.1
−α/ αα thalassemia	1.34	0.84-2.15	0.2
−α/ −α thalassemia	4.30	1.78-10.34	**0.001**
TNF-RI	0.26	0.09-0.70	**0.008**
TNF-RII	2.31	1.08-4.94	**0.03**
sTfR	0.24	0.07-0.63	**0.005**
IL-6	2.05	1.38-3.02	**0.0003**

*Adjusted Odds Ratio. Bold values represent significant differences.

**Table 4:** Multivariate logistic regression analysis of risk factors for fetal anemia in offspring of mothers with OR without iron deficiency.

FACTOR	IRON DEFICIENCY (n = 287)			NO IRON DEFICIENCY (n = 71)		
	aOR*	95% CI	P value	aOR	95% CI	P value
Secundigravidae	1.54	0.72-3.28	0.3	0.13	0.03-0.69	**0.02**
Multigravidae	2.12	1.13-4.00	**0.02**	0.57	0.18-1.82	0.3
−α/ αα	1.68	0.97-2.91	0.1	0.58	0.20-1.69	0.3
−α/ −α	7.02	2.62-18.84	**0.0001**	0.22	0.01-4.24	0.3
TNF-RI	0.19	0.06-0.59	**0.005**	1.10	0.10-10.32	1.0
TNF-RII	2.07	0.90-4.79	0.1	5.64	0.55-57.70	0.1
sTfR	0.12	0.03-0.43	**0.001**	1.68	0.15-18.30	0.7
IL-6	2.60	1.60-4.21	**0.0001**	1.47	0.70-3.12	0.3

*Adjusted Odds Ratio. Bold values represent significant differences.

We therefore conducted analyses after stratifying only the maternal iron deficiency group by parity (**Table 5**). Among all parity groups, fetal anemia risk was higher in newborns with thalassemia, higher cord levels of IL-6, and lower cord levels of TNF-RI and sTfR (**Table 5**). Other than −α/ −α thalassemia, these relationships achieved statistical significance in only some parity groups: increased cord TNF-RI and sTfR significantly reduced fetal anemia risk in first pregnancies, increased cord sTfR significantly reduced fetal anemia risk in second pregnancies, and increased cord IL-6 significantly increased fetal anemia risk in third pregnancies. Of note, reduced sample sizes may limit the power of these subgroup analyses to identify significant relationships.

**Table 5:** Multivariate logistic regression analysis of risk factors for fetal anemia in offspring of mothers with iron deficiency in different parity groups.

FACTOR	PRIMIGRAVIDAE (n = 75)			SECUNDIGRAVIDAE (n = 66)			MULTIGRAVIDAE (n = 146)		
	aOR*	95% CI	P value	aOR	95% CI	P value	aOR	95% CI	P value
−α/ αα	1.68	0.50-5.69	0.4	2.21	0.57-8.59	0.3	1.79	0.85-3.75	0.1
−α/ −α	29.92	1.88-475.28	**0.02**	7.92	1.14-55.17	**0.04**	7.87	1.87-33.10	**0.005**
TNF-RI	0.02	0.001-0.33	**0.006**	0.18	0.01-2.49	0.2	0.23	0.05-1.20	0.1
TNF-RII	3.19	0.37-27.84	0.3	0.96	0.15-6.25	1.0	1.83	0.62-5.40	0.3
sTfR	0.006	0.0001-0.24	**0.007**	0.003	0.0004-0.19	**0.006**	0.40	0.08-1.96	0.3
IL-6	1.92	0.68-5.45	0.2	2.50	0.84-7.41	0.1	2.67	1.33-5.47	**0.006**

*Adjusted Odds Ratio. Bold values represent significant differences.

### Erythropoietin Levels Are Decreased During Severe Fetal Anemia

**Figure 2:**
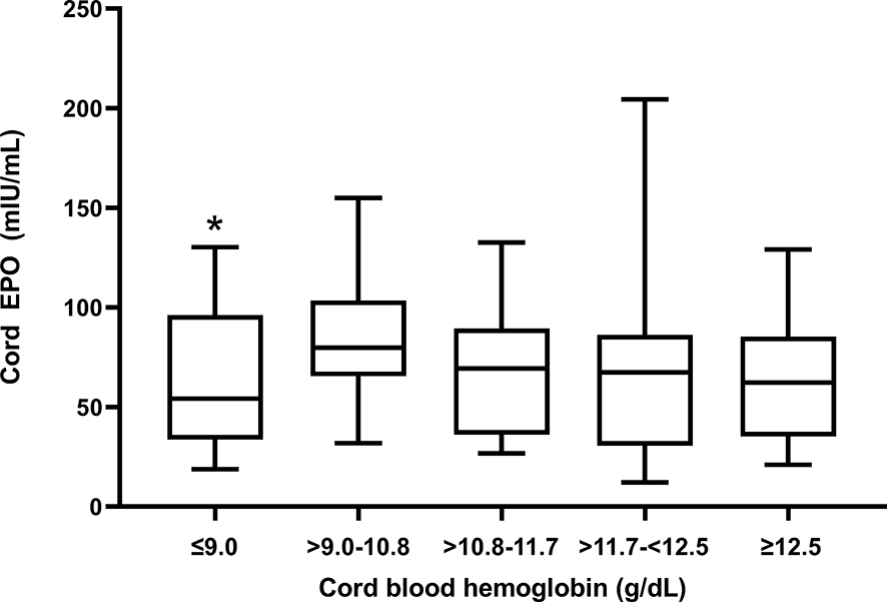
Cord blood EPO levels in relation to degree of fetal anemia. Q1 (Hb ≤ 9 mg/dl); Q2 (Hb > 9 -10.8 mg/dl); Q3 (Hb >10.8-11.7 mg/dl); Q4 (Hb > 11.7-<12.5 mg/dl; and Q5, no fetal anemia (≥12.5). The box plots indicate the median (horizontal line) and interquartile range (box), and the whiskers represent 10^th^/90^th^ percentiles. The differences between groups were analyzed by the Kruskal-Wallis test; *Q1 was significantly lower than the reference group Q5 (p = 0.03).

Cord EPO levels did not differ significantly between anemic (median (interquartile range), 70.8 mIU/mL (35.2 - 85.3)) and non-anemic newborns (62.3 mIU/mL (37.5 – 95.9); p = 0.1, Mann-Whitney test) indicating that EPO is not responding appropriately to declining hemoglobin levels. We stratified anemic newborns in quartiles by fetal hemoglobin levels and compared them to normal newborns for their EPO levels (**Figure 2**). Newborns with fetal anemia showed a non-significant trend to increase EPO as cord hemoglobin decreased, except for newborns in the lowest Hb quartile (Hb ≤ 9.0 g/dl) in whom EPO was significantly lower versus normal children.

## Discussion

Fetal anemia predisposes to anemia in infancy (4) and is a risk factor for infant mortality (5). Fetal anemia is more frequent in malarious areas (6) but the cause is unclear, so we sought to identify maternal and fetal factors that increase the risk of fetal anemia in Tanzania. We confirmed that fetal anemia is common in an area of intense malaria transmission, but that it is not related to PM at the time of delivery. Instead, inappropriately low EPO levels were associated with the most severe fetal anemia in this population. Further, α^+^-thalassemia, sTfR and the cytokine balance (involving TNF-RI, TNF-RII, and IL-6) of the newborn were related to fetal anemia, but these relationships were limited to offspring of iron-deficient mothers in whom all but thalassemia were modified by parity.

We found a high proportion (46.2%) of infants with fetal anemia in our population. The mean cord hemoglobin level of 12.3 g/dl in the present study is lower than levels reported in developing countries without malaria but is comparable to levels from other malarious countries (6). However, placental malaria at delivery was not related to fetal anemia in this study, suggesting that active malaria is not a direct cause. Contradictory findings on the association between malaria in pregnancy and fetal anemia have been reported with some studies showing no association (7–9) but others reporting an association (10–12). Our study suggests that malaria may increase fetal anemia risk through indirect means, such as by causing maternal iron deficiency (44) or by its evolutionary selection of thalassemia (10–12).

Iron is an essential micronutrient for erythropoiesis. The fetus obtains iron from the maternal circulation, and iron deficiency in the mother reduces iron stores in the newborns (45). Recently, animal and human studies have indicated that in the setting of maternal iron deficiency, regulation of placental iron uptake and export to the fetus favors preservation of placental iron sufficiency rather than fetal iron content in order to maintain global function of the healthy placenta (46). We expected that iron deficiency would increase the risk of fetal anemia. However, cord hemoglobin levels did not differ between iron-deficient mothers and mothers with normal iron stores.

Instead, the interaction of maternal iron status and parity was related to cord hemoglobin levels (**Supplementary Table S3**). Fetal anemia prevalence increased significantly with parity when mothers were iron-deficient but tended to decrease when mothers had normal iron stores (**Figure 1**). Maternal iron status modified the relationships of several other factors to fetal anemia. For example, increased levels of cord IL-6 increased the odds and cord TNF-RI decreased the odds of fetal anemia, but only when the mother was iron-deficient. This suggests roles for fetal cytokine balance in the pathogenesis of fetal anemia only during maternal iron deficiency, which suggests that distinct mechanisms converge in the fetus to cause anemia.

Fetal immune activation has been proposed as a mechanism by which placental malaria itself might increase the risk of fetal anemia (6), albeit this and earlier studies (10–12) do not find a direct relationship of placental malaria to fetal anemia. Notably, inflammatory cytokines do not increase in fetal blood during placental malaria (47) and this may explain in part why active placental malaria does not influence the immediate risk of fetal anemia. We cannot rule out that infections prior to delivery may contribute to fetal anemia, as mothers were enrolled in this study at the time of delivery. Future studies should explore whether malaria episodes occurring earlier during pregnancy contribute to the risk of fetal anemia.

The mechanism through which the fetal cytokine milieu may be mediating anemia requires further study. IL-6 is known to induce hypoferremia of infection and inflammation by stimulating the synthesis of hepcidin (48). Hepcidin limits the iron that is available for erythropoiesis by inhibiting its efflux through ferroportin, an iron exporter that is expressed in the small intestine, hepatocytes, macrophages, and placental trophoblast cells (23). Studies to examine IL-6 and hepcidin for their interrelationships to fetal anemia are warranted. Elevated fetal IL-6 was reported in fetal anemia due to maternal Rh alloimmunisation, and presumed to be a marker of fetal systemic inflammatory response syndrome (SIRS) secondary to red blood cell destruction in the reticuloendothelial system (49, 50). Conversely, TNF-RI protects against fetal anemia and might do so by binding and neutralizing TNF-α, an inflammatory cytokine that has been associated with anemia. For this reason, free TNF-α levels in cord blood should be examined in future studies to see whether they have a stronger relationship with fetal anemia than total TNF-α levels.

Maternal iron deficiency and parity also modified the effect of α^+^-thalassemia to cause fetal anemia (**Table 4**). α^+^-thalassemia is the most common hemoglobinopathy of humans and leads to mild hypochromic microcytic anemia. The prevalence of fetal thalassemia in this population (10.1% −α/ −α and 40.1% −α/ αα) is comparable to that found in other malarious areas of Africa (49). Thalassaemia has been related to hematological status of the fetus in a non-malarious area. In New Zealand, cord levels of hemoglobin, mean corpuscular hemoglobin (MCH) and mean corpuscular volume (MCV) were significantly lower in heterozygous and homozygous thalassemic newborns compared to normal newborns (51, 52).

In the present study, decreased sTfR levels were associated with increased odds of fetal anemia. Erythropoietic activity has been found to be the most important determinant of sTfR levels (51, 53). In conditions such as α-thalassemia which are characterized by stimulated erythropoiesis, sTfR levels are increased; elevated levels of sTfR in older children with thalassemia were previously reported from Vanuatu where malaria is endemic (54). Elevated sTfR may reflect stimulated erythropoiesis in the fetus with thalassemia and anemia.

EPO levels were not significantly increased in newborns with fetal anemia, and this inappropriately blunted response may have contributed to anemia in our study population. EPO is the primary growth factor regulating red blood cell production, and increased cord blood EPO levels are a marker of chronic fetal hypoxia and intrauterine fetal growth retardation (55), as well as Rh isoimmunization (28). EPO does not cross the placenta (56) and therefore must emanate from fetal sources, such as kidneys, liver, and placenta which are known to express the EPO gene (57). We speculate that the degree of fetal anemia observed in Tanzanian newborns may not cause sufficient hypoxia to induce EPO production. Rodent studies suggest that the EPO response to hypoxia is markedly reduced in newborn compared to adult rats (58), possibly because the high oxygen-affinity fetal hemoglobin mitigates tissue hypoxia due to anemia. Alternatively, TNF-α and IL-1 have been shown to inhibit EPO production (59) and therefore the inflammatory environment could suppress EPO production by the fetus during anemia. Indeed, EPO levels were significantly decreased in newborns with the most severe anemia (**Figure 2**), suggesting that a failed EPO response might actively contribute to fetal anemia.

In summary, this study has shown that fetal α-thalassemia, cytokine balance, and sTfR levels, but not PM, are related to fetal anemia risk in an area of high malaria transmission. The relationship of these factors to fetal anemia was only seen when mothers were iron-deficient, suggesting that measures to prevent maternal iron deficiency may reduce fetal anemia risk, albeit maternal iron status was not directly related to fetal anemia. The opposing effects of inflammatory (IL-6) and anti-inflammatory (soluble TNF-RI) factors suggest that conditions that alter fetal cytokine balance may be involved in the pathogenesis of fetal anemia. Future studies are needed to elucidate the causes of altered fetal cytokine balance and their roles in the pathogenesis of fetal anemia. Inadequate EPO may also be a contributor to the most severe cases of anemia in Tanzanian newborns.

## Data Availability Statement

All relevant data are included in the manuscript and are available from the authors upon reasonable request and execution of inter-institutional agreements for sharing of human data.

## Ethics Statement

The studies involving human participants were reviewed and approved by the Division of Microbiology and Infectious Diseases at the U.S. National Institutes of Health and by the institutional review boards of Seattle BioMed and the Medical Research Coordinating Committee in Tanzania. Written informed consent to participate in this study was provided by the participants’ legal guardian/next of kin.

## Author Contributions

PD and MF designed the MOMS Project. JK and EK completed the cytokine assays. GM determined variants of red cell abnormalities. AM performed histological studies. EK and PD analyzed the data and wrote the manuscript with the contribution of all other authors. All authors contributed to the article and approved the submitted version.

## Funding

This work was supported by the Intramural Research Program of the National Institute of Allergy and Infectious Diseases, National Institutes of Health, and by grants from the Bill & Melinda Gates Foundation (Grant 29202), NIH (R01 AI 52059) and the Fogarty International Center/NIH (TW 05509) to PD. PD and MF are supported by the Intramural Research Program of NIAID, NIH.

## Conflict of Interest

The authors declare that the research was conducted in the absence of any commercial or financial relationships that could be construed as a potential conflict of interest.
