# Supplementary material for: Fetal Cytokine Balance, Erythropoietin and Thalassemia but Not Placental Malaria Contribute to Fetal Anemia Risk in Tanzania
Source: Front Immunol. 2021 Apr 30;12:624136. doi: 10.3389/fimmu.2021.624136 (PMC8120033; doi:10.3389/fimmu.2021.624136)
Supplement: Supplementary file 1 [file Presentation_1.pdf]

Supplementary Figure S1. Approach to analysis of risk factors for fetal anemia

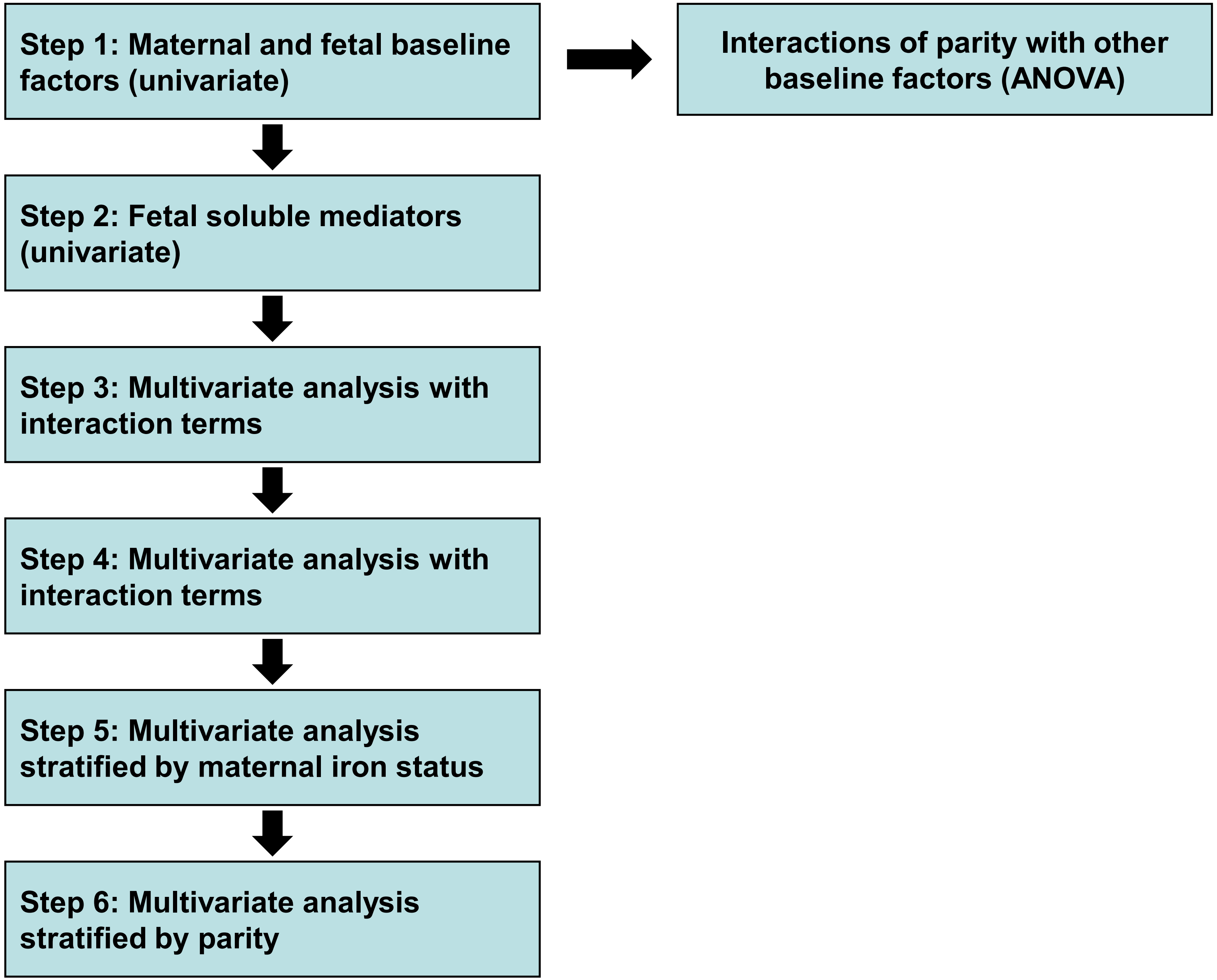

Supplementary Table S1: Fetal anemia was more frequent in the PM+ than the PM- group in each parity category using cord hemoglobin < 12.5 g/dl

Primigravidae

	No PM	Yes PM	P value
No fetal anemia	78/127 (61.4%)	24/40 (60.0%)	0.87
Yes fetal anemia	49/127 (33.3%)	16/40 (40.0%)	

Secundigravidae

	No PM	Yes PM	P value
No fetal anemia	69/117 (58.9%)	16/29 (20.7%)	0.71
Yes fetal anemia	48/117 (41.0%)	13/29 (44.8%)	

Multigravidae

	No PM	Yes PM	P value
No fetal anemia	133/276 (48.2%)	8/21(38.1%)	0.37
Yes fetal anemia	143/276 (51.8%)	13/21(61.9%)	

Supplementary Table S2: Fetal anemia was more frequent in the PM+ than the PM- group in each parity category using cord hemoglobin < 10.0 g/dl

Primigravidae

	**No PM**	**Yes PM**	**P value**
No fetal anemia	106/127 (83.4%)	31/40 (77.5%)	0.40
Yes fetal anemia	21/127 (16.5%)	9/40 (22.5%)	

Secundigravidae

	**No PM**	**Yes PM**	**P value**
No fetal anemia	107/117 (91.4%)	25/29 (86.2%)	0.40
Yes fetal anemia	10/117 (8.6%)	4/29 (13.8%)	

Multigravidae

	**No PM**	**Yes PM**	**P value**
No fetal anemia	223/276 (80.8%)	15/21 (71.4%)	0.32
Yes fetal anemia	53.276 (10.2%)	6/12 (28.6%)	

Supplementary Table S3. Relationships of cord hemoglobin levels to interactions between parity and baseline maternal and newborn factors.

INTERACTION TERM	P value
Parity * Maternal iron deficiency	**0.03**
Parity * PM (microscopy)	0.7
Parity * Maternal anemia	0.2
Parity * PM (histology)	0.5
Parity * Newborn sickle hemoglobin types	0.4
Parity * Newborn α−thalassemia	0.5
Parity * Newborn iron deficiency	0.9
Parity * Maternal age	0.5
Parity * Birth weight	0.4
Parity * Infant sex	0.5

Supplementary Table S4. The frequency of fetal iron deficiency did not differ between newborns with or without fetal anemia (Cord hemoglobin < 10.0 g/dl)

	No fetal iron deficiency	Yes fetal iron deficiency	P value
No fetal anemia	404/483 (83.6%)	60/73 (82.2%)	0.75
Yes fetal anemia	79/483 (16.450	13/73 (17.8%)	

Supplementary Table S5. The frequency of maternal iron deficiency did not differ between newborns with or without fetal anemia (Cord hemoglobin < 10.0 g/dl)

	No maternal iron deficiency	Yes maternal iron deficiency	P value
No fetal anemia	96/111 (86.5%)	379/461 (82.2%)	0.28
Yes fetal anemia	15/111 (13.5%)	82/461 (17.8%)	

Supplementary Table S6. The frequency of neonatal genotypes/phenotypes did not vary on the basis of PM status or parity.

Thalassemia and Parity

	Primigravid	Secundi	Multigravid	P value
Normal	93/183 (50.8%)	74/148 (50.0%)	148/301 (49.2 %)	0.61
−α/ αα	77/183 (42.1%)	57/148 (38.5%)	119/301 (39.5 %)	
−α/ −α	13/183 (7.1%)	17/148 (11.5%)	34/301 (11.3%)	

Thalassemia and PM

	PM-	PM+	P value
Normal	273/545 (60.1%)	42/87 (48.3%)	0.43
−α/ αα	214/545 (39.3%)	39/87 (44.8%)	
−α/ −α	58/545 (10.6%)	6/87 (6.9%)	

G6PD and parity

	Primigravid	Secundigravid	Multigravid	P value
A	25.7%	26.7%	47.6%	0.52
A-	20.9%	24.4%	54.6%	
A heterozygous	100.0%	0.0%	0.0%	
A- heterozygous	27.4%	15.7%	56.9%	
B	30.8%	21.9%	47.3%%	

G6PD and PM

	PM -	PM+	P value
A	89.1%	10.9%	0.47
A-	88.4%	11.6%	
A heterozygous	100.0%	0.0%	
A- heterozygous	90.2%	9.8%	
B	87.6%	12.4%	

Neonatal hemoglobin types and parity

Hb Type	Primigravid	Secundigravid	Multigravid	P value
AA	82.4%	83.4%	82.0%	0.92
AS	16.7%	15.5%	17.4%	
SS	0.9%	1.1%	0.6%	

Neonatal hemoglobin types and PM

Hb Type	PM-	PM+	P value
AA	82.9%	80.0%	0.29
AS	16.6%	18.0%	
SS	0.5%	2.0%	
